# *CanDrivR-CS*: a cancer-specific machine learning framework for distinguishing recurrent and rare variants

**DOI:** 10.1093/bioadv/vbag008

**Published:** 2026-01-12

**Authors:** Amy Francis, Colin Campbell, Tom R Gaunt

**Affiliations:** MRC Integrative Epidemiology Unit, Bristol Medical School (PHS), University of Bristol, Oakfield House, Bristol BS8 2BN, United Kingdom; Intelligent Systems Laboratories, University of Bristol, Bristol BS1 5DD, United Kingdom; MRC Integrative Epidemiology Unit, Bristol Medical School (PHS), University of Bristol, Oakfield House, Bristol BS8 2BN, United Kingdom

## Abstract

**Motivation:**

Missense variants—single nucleotide substitutions that result in an amino acid change in the encoded protein—play an important role in cancer. Distinguishing between recurrent and rare missense variants may reveal insights into selective pressures and functional consequences. While recurrent variants may undergo positive selection across patients, rare variants can also drive resistance or other phenotypes. However, most existing tools predict pathogenicity across broad populations and ignore tumour-specific contexts. Here, we present *CanDrivR-CS*, a suite of cancer-specific gradient boosting models designed to distinguish between rare and recurrent somatic missense variants.

**Results:**

We curated data from the International Cancer Genome Consortium (ICGC) and trained 50 cancer-specific models. These significantly outperformed a pan-cancer baseline, achieving up to 90% F1 score in leave-one-group-out cross-validation (LOGO-CV) for skin melanoma. Notably, DNA shape features ranked among the most predictive across all cancers, with recurrent variants enriched in structurally complex DNA regions such as bends and rolls—potential mutational hotspots.

**Availability and implementation:**

All code and data are available at *CanDrivR-CS* GitHub repository https://github.com/amyfrancis97/CanDrivR-CS, with further advice on the installation procedure in Section 1 of the Supplementary Materials.

## 1 Introduction 

Recent advancements in next-generation sequencing have revolutionised the discovery of human genetic variants, revealing their prevalence across diverse genetic backgrounds in both health and disease contexts (Park *et al.* 2019, Alexandrov *et al.* 2020, Zhao *et al.* 2021, Solis-Moruno *et al.* 2023). Despite progress in identifying these variants, understanding their biological implications and contributions to disease phenotypes remains a significant challenge. Gaining further insights into their roles in disease is, therefore, crucial for advancing drug development and precision medicine.

In recent years, significant efforts have been made to develop methods for interpreting the role of Single Nucleotide Variants (SNVs). Functional assays, such as Multiplexed Assays of Variant Effect (MAVE), are increasingly being used for this purpose (Weile and Roth 2018, Chiasson *et al.* 2019, Nielsen *et al.* 2021, Hoskins *et al.* 2023). MAVE assays systematically introduce genetic alterations and measure their impact on cellular phenotypes, including growth, gene expression, and protein function. In parallel with experimental methodologies, various software tools have been proposed for predicting the downstream consequences of variants in the genome. Thus, recently proposed methods use extended sets of molecular-level features (Yang *et al.* 2025), protein-level effects, such as protein surface features (Zhou *et al.* 2025), or protein-structure graph features (Danner *et al.* 2025) to understand effects on proteins.

However, the vast size of the human genome results in a combinatorial explosion, creating substantial challenges in terms of scalability, labour intensity, and cost. Consequently, there is an urgent need for complementary tools to assist in interpreting and predicting the effects of novel genetic variants within disease contexts.

Various computational tools have emerged to predict the pathogenic consequences of SNVs. Widely used tools such as *PolyPhen-2* (Adzhubei *et al.* 2013), *SIFT* (Ng and Henikoff 2003), and *MutationTaster* (Schwarz *et al.* 2010) assess the potential impact of SNVs across various diseases. However, these tools often lack specificity to variants that drive particular diseases, instead predicting pathogenic consequences across all disease genomes. Thus our *CScape* predictor citepshihab2015 focused on predicting SNV impacts in cancers (Rogers *et al.* 2017), yet without differentiation among cancer types.

In addition to understanding how mutational landscapes differ between cancer types, further insights could be gained by investigating why many cancer variants are rare while others occur more frequently. For example, rare secondary mutations can arise as mechanisms of resistance to treatment, such as the secondary resistance mutations that develop in the EGFR gene in response to Gefitinib treatment (Pao *et al.* 2005). Furthermore, common mutations may play a critical role in cancer development and progression, potentially undergoing positive selection across multiple individuals due to the growth advantage they confer to tumour cells, driving cancer phenotypes (Vogelstein *et al.* 2013, Martincorena 2019).

**Figure 1:**
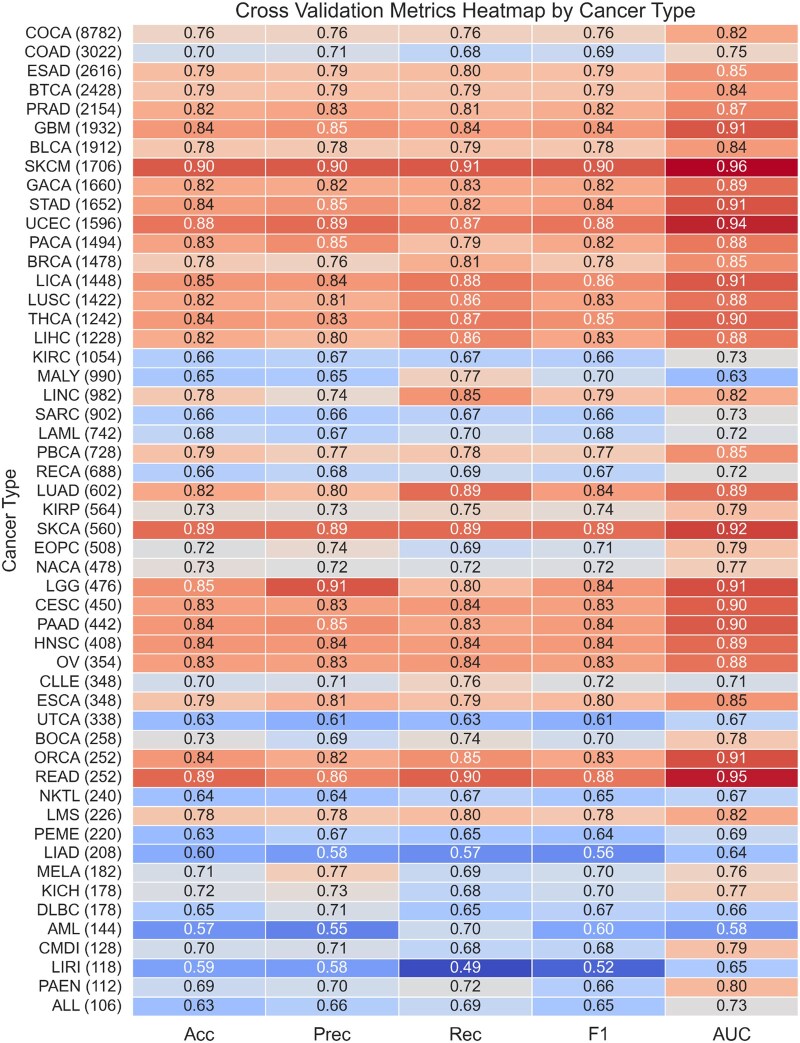
Cross-validation results heatmap sorted by total dataset size, with datasets below 100 samples excluded. This table is also presented in Section 2.6, available as supplementary data at *Bioinformatics Advances* online in alphabetic order by cancer type. Cancer datasets exceeding 1000 samples consistently lead to high predictive performance. Notably, UCEC, SKCM, and READ emerge as top performers, achieving F1 scores of 88%, 90%, and 88%, respectively. Despite their smaller sizes, datasets like SKCA also performed well.

This paper introduces *CanDrivR-CS*, a collection of cancer-specific machine learning algorithms that are designed to distinguish between recurrent and rare SNVs, trained on data from the International Cancer Genome Consortium (ICGC) (Hudson *et al.* 2010). The methodology for classifying variants was inspired by our previous work for *CScape-Somatic* (Rogers *et al.* 2020). Our findings suggest that building tailored machine learning models that consider the disease context of a variant can increase the performance by up to 11% (see Table 3 and Fig. 1), compared to taking a pan-cancer prediction approach.

## 2 Materials and methods

The following section outlines the methods used in this work. For further descriptions, please refer to our GitHub repository and the Supplementary, available as supplementary data at *Bioinformatics Advances* online.

### 2.1 Preparation of train and test data

All datasets were prepared by filtering to autosomal, single nucleotide missense variants. Missense variants—single base substitutions that result in amino acid changes—were selected as the focus of this study due to their functional relevance in cancer and the comparatively rich set of available feature annotations, including structural, evolutionary, and biochemical properties. In contrast, synonymous variants and those on sex chromosomes exhibit distinct biological characteristics and annotation limitations, and would be more appropriately addressed in a separate, dedicated analysis. A summary of the training and test datasets used in this analysis is provided in Table 1.

**Table 1:** This table shows a summary of the train and test datasets used in this analysis.

Data	Variant type	Data type	No. variants
**ICGC**	Somatic	Train/Test	135 648
**COSMIC**	Somatic	Test	240 894
**TCGA-UCEC**	Somatic	Test	557
**TCGA-SKCM**	Somatic	Test	103

#### 2.1.1 Training data: the international cancer genome consortium (ICGC)

**Downloading ICGC Data:** We downloaded V1.0 of the ‘simple_somatic_mutation.aggregated.vcf.gz’ file from the International Cancer Genome Consortium (ICGC) website (Hudson *et al.* 2010). We updated the genomic coordinates from the hg19 genome build to the hg38 genome build using *pyliftover* in Python, facilitated by the hg19ToHg38 chain file from the UCSC Genome Browser (Nassar *et al.* 2023).

**Preparation of ICGC Data:** In this work, we propose two models. The first model, *CanDrivR*, serves as our baseline model. This model takes a pan-cancer approach; designed to distinguish between recurrent and rare somatic mutations across all cancer types included in the ICGC dataset.

Building upon the baseline, we introduce *CanDrivR-CS*, an ensemble of cancer-specific machine learning models tailored to each individual cancer type within ICGC.

***CanDrivR Baseline Model:*** For our baseline model, our rare class was defined as variants occurring in exactly one patient in the ICGC dataset. To establish the recurrent class, we experimented with training our model using different donor count thresholds (Fig. 1, available as supplementary data at *Bioinformatics Advances* online). *CanDrivR’s* performance was highest when using a donor count threshold of > 2 for the recurrent class. After applying this threshold, we identified 67 824 variants in the recurrent class and 2 035 826 variants in the rare class. To balance the data, we randomly down-sampled the rare class to match the number of recurrent variants. Hence, our final dataset contained 135 648 missense variants.

**Figure 2:**
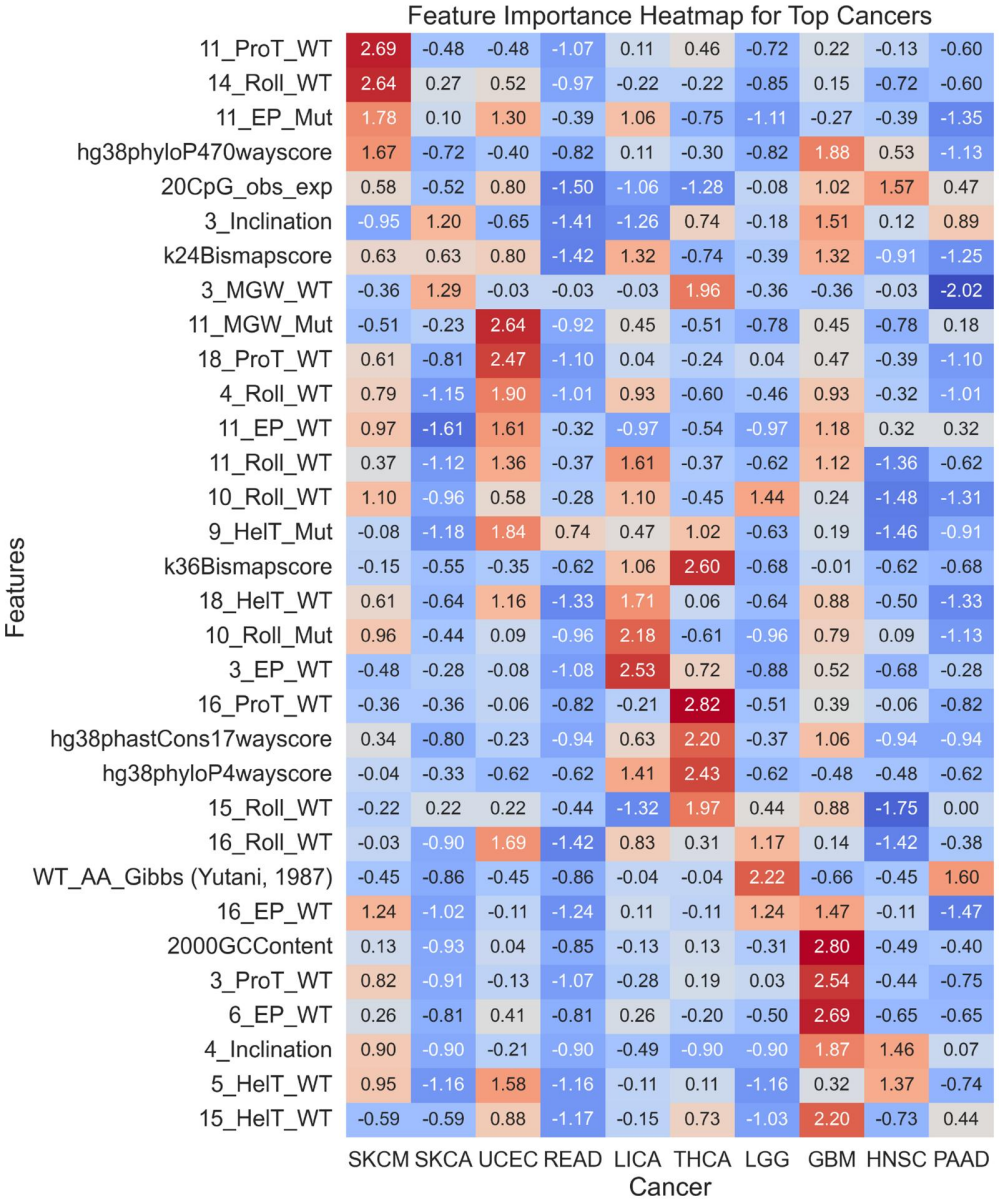
In this figure, we calculated the feature importance for the top five features for each of the cancer types. The feature importance was calculated using XGBoost’s get_booster().get_score() function, and then the data was scaled using Scikit Learn’s StandardScaler() for plotting. For each of the DNA shape features (e.g. Propellar Twist (ProT), Helix Twist (HelT), Electrostatic Potential (EP), and Roll), the number proceeding the feature type is the position at which the value is recorded. For example, a position of ‘11’ indicates the site of the nucleotide variant, a position of ‘10’ indicates the nucleotide position to the left of the variant, and ‘12’ indicates the nucleotide immediately to the right of the variant. For more information on DNA shape features, please see Tables 2–7, available as supplementary data at *Bioinformatics Advances* online.

***CanDrivR-CS Model:*** After developing our baseline model, we developed *CanDrivR-CS* by training a separate model for each unique cancer type within ICGC. To keep the sizes of our datasets large enough to model, we pooled cancer datasets across different study sub-populations. In *CanDrivR-CS* models, the rare class was defined in the same way as our baseline model; using variants with a donor count of exactly one. For the recurrent class, we optimised the donor count threshold for each cancer model. To determine these thresholds, we produced optimisation curves (see Fig. 2, available as supplementary data at *Bioinformatics Advances* online for some examples) similar to those in the baseline model (Fig. 1, available as supplementary data at *Bioinformatics Advances* online). This approach allowed us to tailor each model to the specific mutation recurrence patterns observed in each type of cancer. We then down-sampled the majority class and removed any cancer types that had a balanced dataset of <100 samples, leading to 50 different cancer models. The thresholds and total dataset sizes used for training each of the *CanDrivR-CS* models are detailed in Table 2.

**Table 2:** Cancer types, donor counts, and sizes of balanced datasets for the 50 cancer types considered.^a^

Cancer name	Cancer code	Donor count threshold (>)	Size
**Acute lymphoblastic leukemia**	ALL	1	106
**Acute myeloid leukemia**	AML	3	144
**Bladder urothelial carcinoma**	BLCA	2	1912
**Bone cancer**	BOCA	2	258
**Breast cancer**	BRCA	3	1478
**Biliary tract cancer**	BTCA	2	2428
**Cervical squamous cell carcinoma**	CESC	3	450
**Chronic lymphocytic leukemia**	CLLE	2	348
**Colon adenocarcinoma**	COAD	1	3022
**Colorectal cancer**	COCA	6	8782
**Diffuse large B-cell lymphoma**	DLBC	2	178
**Early onset prostate cancer**	EOPC	2	508
**Esophageal adenocarcinoma**	ESAD	3	2616
**Esophageal carcinoma**	ESCA	4	348
**Gastric cancer**	GACA	3	1660
**Glioblastoma multiforme**	GBM	3	1932
**Head and neck squamous cell carcinoma**	HNSC	2	408
**Kidney chromophobe**	KICH	1	178
**Kidney renal clear cell carcinoma**	KIRC	1	1054
**Kidney renal papillary cell carcinoma**	KIRP	2	564
**Liver adenocarcinoma**	LIAD	1	208
**Liver intrahepatic cholangiocarcinoma**	LICA	3	1448
**Liver hepatocellular cancer**	LIHC	2	1228
**Liver cancer**	LIRI	1	118
**Lower grade glioma**	LGG	3	476
**Lung adenocarcinoma**	LUAD	3	602
**Liver cancer**	LINC	2	982
**Lung squamous cell carcinoma**	LUSC	3	1422
**Leiomyosarcoma**	LMS	2	226
**Malignant lymphoma**	MALY	1	990
**Melanoma**	MELA	2	182
**Nasopharyngeal cancer**	NACA	1	478
**Natural killer/T-cell lymphoma**	NKTL	1	240
**Oral cancer**	ORCA	2	252
**Ovarian cancer**	OV	3	354
**Pancreatic adenocarcinoma**	PAAD	3	442
**Pancreatic cancer endocrine neoplasms**	PAEN	2	112
**Pancreatic cancer**	PACA	2	1494
**Pediatric brain cancer**	PBCA	2	728
**Pediatric medulloblastoma**	PEME	2	220
**Prostate cancer**	PRAD	2	2154
**Rectum adenocarcinoma**	READ	6	252
**Rectal cancer**	RECA	1	688
**Sarcoma**	SARC	1	902
**Skin adenocarcinoma**	SKCA	5	560
**Skin cutaneous melanoma**	SKCM	4	1706
**Stomach adenocarcinoma**	STAD	3	1652
**Thyroid carcinoma**	THCA	4	1242
**Uterine carcinosarcoma**	UTCA	1	338
**Uterine corpus endometrial carcinoma**	UCEC	3	1596

a This table details the cancer codes used to build models based on the ICGC dataset, the thresholds selected for the recurrent training dataset in *CanDrivR-CS*, and the final column shows the total size of the balanced dataset used for training and testing. The definitions of cancer codes are derived from several sources (Hudson *et al.* 2010, Weinstein *et al.* 2013, Guo *et al.* 2018, Nassar *et al.* 2023). Figure 1 additionally states results for two myeloid disorders with typecodes LAML (Acute Myeloid Leukemia) and CMDI (Chronic Myeloid Disorders): the corresponding datasets were studied separately to ensure clinical phenotype labelling integrity.

**Table 3:** Cross-validation and test results for rare (donor count = 1) versus recurrent (donor count > 2) variants.^a^

Dataset	Result	Acc. (%)	Prec. (%)	Rec. (%)	F1 (%)	AUC (%)
**ICGC**	Cross-Val	78.9 ± 1.5	78.4 ± 1.7	80.0 ± 2.3	79.2 ± 1.7	85.2 ± 1.8
**ICGC**	Test	80.1	78.3	80.8	79.6	79.8
**COSMIC**	Test	66.5	64.5	66.7	65.6	65.0

a The cross-validation metrics represent the mean results for the cross-validation folds, and the standard deviations represent the standard deviations across the folds. The test statistics represent the held-out test data, and unseen COSMIC data.

#### 2.1.2 Baseline model test data: the catalogue of somatic mutations in cancer (COSMIC)

We evaluated our *CanDrivR* baseline model on rare and recurrent mutations from the Catalogue of Somatic Mutations in Cancer (COSMIC) (Tate *et al.* 2019). Specifically, we downloaded v.99 of the COSMIC ‘genome screens mutant’ file in GRCh38 format. We then removed any variants present in our ICGC training data, filter to *r* = 1 and *r* > 2, and down-sample the majority group. We remove all variants present in our training data. In total, this led to a dataset size of 240 894 variants.

#### 2.1.3 Cancer-specific test data: the cancer genome atlas (TCGA)

To evaluate our top-performing cancer-specific models, we used data from The Cancer Genome Atlas (TCGA) (Weinstein *et al.* 2013), selecting models with sufficient samples (over 100 after removing variants present in our training data). TCGA was chosen for its consistent cancer labelling with ICGC, enabling straightforward subgrouping by cancer type; minimising any bias. Using the *TCGAbiolinks* package (Colaprico *et al.* 2016) in R, we downloaded TCGA data. We created unique mutation identifiers, and counted distinct tumour sample barcodes for each mutation. We focused on uterine corpus endometrial carcinoma (UCEC) and skin cutaneous melanoma (SKCM), as these were the only cancers with over 100 variants after excluding ICGC training data. To create balanced datasets, we filtered mutations by sample count thresholds (>4 for SKCM and >3 for UCEC) and randomly selected an equal number of mutations with a sample count of one, matching the thresholds in the respective *CanDrivR-CS* training data. This resulted in final dataset sizes of 103 variants for SKCM and 557 variants for UCEC.

### 2.2 Algorithms and hyperparameters

We used the XGBClassifier() function from the XGBoost library (version 1.7.6) in Python (Chen and Guestrin 2016), based on its high performance and efficiency in prior evaluations. The rationale for this selection was supported by our previous research, where we systematically compared multiple classifiers and found XGBoost to consistently outperform alternative methods across similar variant prediction tasks (Rogers *et al.* 2020). To ensure full reproducibility, all models were trained using a fixed random state of 42, including both the pan-cancer *CanDrivR* baseline and the 50 cancer-specific *CanDrivR-CS* models.

To maintain comparability across tumour types and to prioritise methodological consistency, we adopted the default hyperparameters provided by XGBClassifier (e.g. learning rate = 0.3, max depth = 6, 100 estimators). This decision reflects a deliberate focus on evaluating model architecture, feature design, and cross-cancer performance trends, rather than fine-tuning individual models for absolute peak performance.

We note that hyperparameter optimisation may yield modest gains in predictive performance, particularly for under-represented cancer types. However, due to the computational demands of training and evaluating 50 independent models, we opted to prioritise interpretability and consistency. Future work could explore targeted hyperparameter tuning or automated optimisation frameworks (e.g. Optuna, Hyperopt) to refine performance further, particularly in low-data or high-variance settings.

### 2.3 Handling missing data

We quantified feature-level missing values across the dataset. Most features had high coverage, with the majority of feature sets having missing values for fewer than 10% of variants. In all cases, we assume MCAR (value missing completely at random), since any assumption otherwise would be to assume insight into the data gathering experimentation and knowledge of missing value dependence on observed or unobserved data values. Features with greater proportions of missing values, such as multi-species conservation scores (e.g. phyloP, phastCons), reflect incomplete genomic annotations at certain loci. A summary of such MCAR missing percentages is provided in Table 1, available as supplementary data at *Bioinformatics Advances* online. To handle missing values, we relied on XGBoost’s internal sparsity-aware algorithm (Chen and Guestrin 2016). This method automatically learns the optimal direction for missing values during tree construction by evaluating both branches at each split and selecting the one that maximises information gain. As such, we did not apply external imputation.

### 2.4 Features

**Figure 3:**
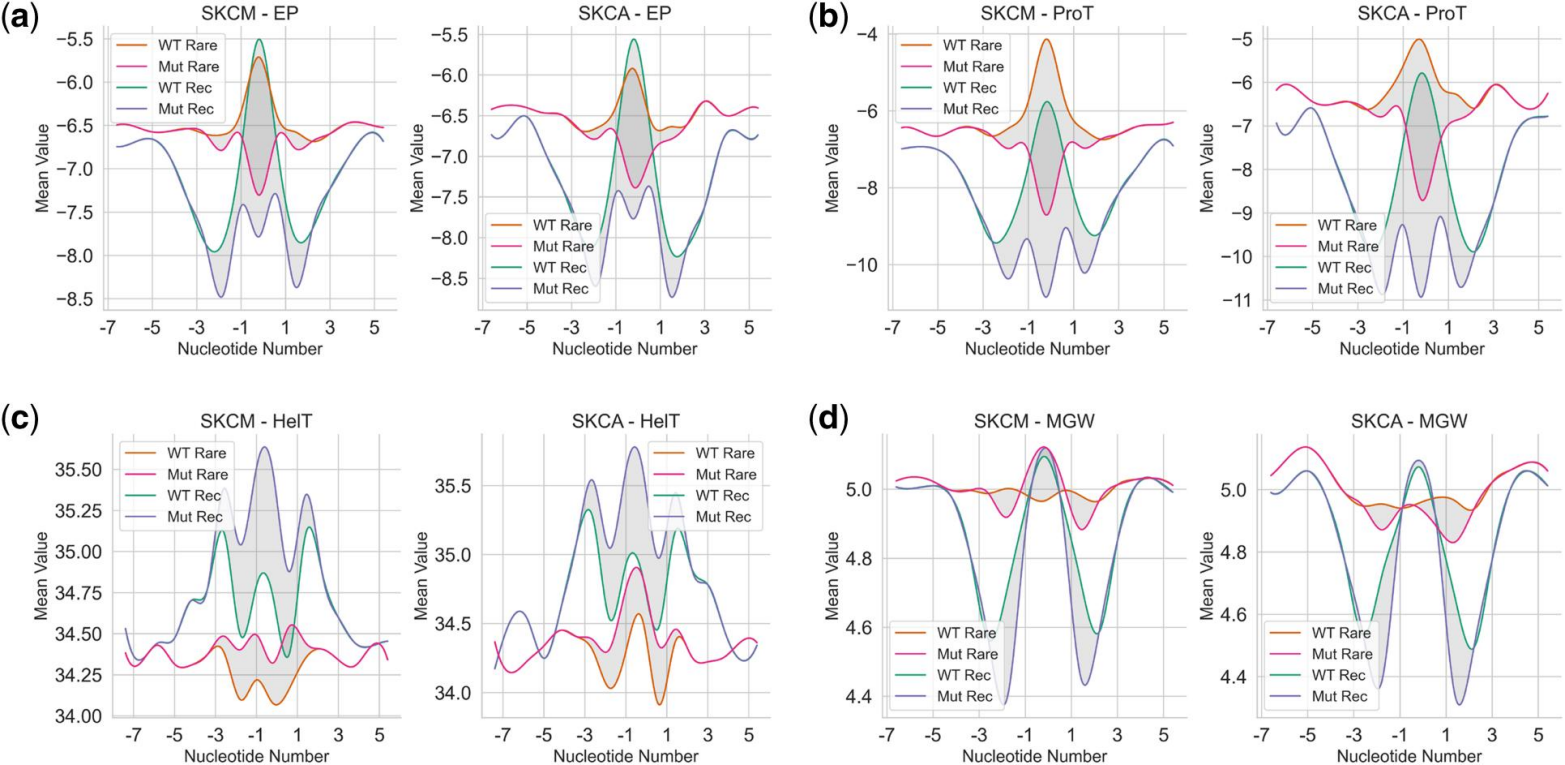
DNA shape results. Using DNAShapR, we predict the (a) electrostatic potential (EP), (b) propeller twist (ProT), (c) helix twist (HT), and (d) minor groove width (MGW) for nucleotide positions −7 to +7 flanking either side of the single nucleotide variant. Position 0 denotes the location of the substitution. As seen in previous figures, this feature is denoted as ‘11_feature.’ We capture DNA shape values for both the wild-type DNA sequence and the mutant sequence. For both the ‘rare’ (r = 1) and the ‘recurrent’ dataset (r > n, where ‘n’ is the threshold value specific to different cancer types), we plot the mean value of all variants for each DNA shape feature and position. The figure presents results for SKCM and SKCA datasets. Recurrent variants tend to occur in regions characterised by high flexibility, bends, and twists, while rare variants are observed in comparatively ‘flatter’ regions. In both classes, there is a shift in DNA properties after the substitution, and this impact regions flanking the substitution site.

We extracted features using the *DrivR-Base* framework (Francis *et al.* 2024). For an in-depth description of the features and their sources, please refer to our Supplementary Material, available as supplementary data at *Bioinformatics Advances* online, and our previous *DrivR-Base* paper (Francis *et al.* 2024). In this work, we used sequential feature selection to retain the most informative features (see Fig. 3, available as supplementary data at *Bioinformatics Advances* online for more details). Our feature selection led to a final feature set of 351 features. A full list of selected features can be found in Tables 2–7, available as supplementary data at *Bioinformatics Advances* online.

**Table 4:** Performance metrics for *CanDrivR-UCEC* and *CanDrivR-SKCM* models on ‘seen’ (ICGC Test) and ‘unseen’ (TCGA) datasets.^a^

Cancer	Dataset	Acc. (%)	Prec. (%)	Rec. (%)	F1 (%)	AUC (%)
**UCEC**	ICGC	86.6	87.6	85.6	86.5	93.3
**UCEC**	TCGA	81.9	82.1	76.2	79.0	82.0
**SKCM**	ICGC	87.1	84.7	89.3	86.9	87.0
**SKCM**	TCGA	87.9	93.8	90.0	91.8	87.9

a Results include accuracy, precision, recall, F1 score, and AUC score.

The selected features encompass a wide range of biological properties, and can be broadly categorised into the following feature groups:

1. **Conservation-based scores**: These scores measure the evolutionary conservation of nucleotide sequences, indicating the likelihood of a variant being deleterious if it occurs in a highly conserved region (Siepel *et al.* 2005, Raimondi *et al.* 2021).
2. **Variant Effect Predictor consequences and amino acid prediction**: This group includes the predicted impact of variants, such as the introduction of stop codons (McLaren *et al.* 2016).
3. **Dinucleotide properties**: Features related to the physical and chemical properties of dinucleotide sequences, which can influence DNA stability (Friedel *et al.* 2009).
4. **DNA shape properties**: Structural properties of DNA, such as minor groove width and helix twist, which can affect how DNA interacts with proteins and other molecules (Chiu *et al.* 2016).
5. **GC/CpG content**: The proportion of guanine-cytosine pairs and the presence of CpG islands, which are regions rich in CG dinucleotides and can be associated with gene regulatory elements.
6. **Kernel-based sequence similarity**: Computational measures that measure the similarity between sequences using kernel-based methods (Campbell and Ying 2011).
7. **Amino acid substitution matrices**: Matrices that provide scores for the likelihood of one amino acid being substituted for another, based on evolutionary data (e.g. BLOSUM, PAM) (Pelé *et al.* 2012).
8. **Amino acid properties**: Features that describe the physical, chemical, and functional properties of amino acids, such as hydrophobicity, charge, and molecular weight (Kawashima and Kanehisa 2000).

### 2.5 Model evaluation

All models were optimised and evaluated using leave-one-group-out cross-validation (the definition of a *group* is given below). The rationale behind this choice was to prevent data leakage during the evaluation process. If we were to randomly partition samples into training and validation sets, it might result in training and test variants that are in close proximity and therefore share very similar features. This would not provide a true reflection of the model’s ability to generalise to unseen data. To mitigate this issue, we divided the data into 11 groups, with each group containing variants from two randomly assigned chromosomes. This ensured that our validation and test data remained distinct from our training dataset:

1. We randomly split our data into 11 groups, each containing variants from two distinct chromosomes (e.g. chr1 and chr5 variants assigned to group 1).
2. Out of these 11 groups, we then randomly selected a single group to hold out for testing.
3. From the remaining training/validation dataset, we held out a second group for validation. We trained our model using the remaining nine groups and validated our model using this held-out validation dataset.
4. We calculated the accuracy, precision, recall, F1, and area under the curve (AUC) scores, using XGBoost’s built in ‘metrics’ function.
5. We repeated this cross-validation process ten times, each with a different validation dataset.
6. Our final *validation* result is the mean of all metrics over all folds of cross-validation.
7. We then re-trained our model using all ten training/validation groups and tested on the 11th group. These results are defined as our *test* results.

## 3 Results

### 3.1 Baseline model

#### 3.1.1 Evaluating on ICGC data

We built our baseline gradient boosting model, *CanDrivR*, using pan-cancer variants from ICGC (Hudson *et al.* 2010). As detailed in our Methods section, we evaluated our model using leave-one-group-out cross-validation (LOGO-CV). The mean metrics over all folds of cross-validation, along with the overall test performance, are presented in Table 3. *CanDrivR* was able to generalise very well across all chromosomes (cross-validation F1 = 79.2 ± 1.7%) and the held out test set (F1 = 79.6%).

#### 3.1.2 Evaluation on unseen COSMIC data

Next, we evaluated our baseline model using data from COSMIC (Tate *et al.* 2019). Our baseline model was unable to generalise well to this unseen dataset, and only achieved an F1 score of 65.6% (Table 3). We discuss possible explanations for this observation in the Discussion.

### 3.2 Cancer-Specific models

#### 3.2.1 Evaluation

Following our baseline model, we developed *CanDrivR-CS*, a cancer-specific gradient boosting framework. We partitioned the ICGC dataset into smaller subsets based on cancer type and constructed distinct models for each of these. *CanDrivR-CS* consistently achieved markedly higher cross-validation performance, with the top models reaching an F1 score of 90% (Fig. 1), compared to the baseline model’s F1 score of 79.2%. Notably, *CanDrivR-SKCM (Skin Cutaneous Melanoma)* and *CanDrivR-SKCA (Skin Adenocarcinoma)* were among the highest performers, achieving F1 cross-validation scores of 90% and 89%, respectively.

Furthermore, datasets exceeding 1000 samples consistently achieved the highest performance, with most F1 scores surpassing 79%. In contrast, smaller datasets containing fewer than 1000 variants showed greater variability. For instance, *CanDrivR-LIRI (Liver Cancer)* yielded an F1 score of 52%, with a dataset size of 118 variants. However, there were exceptions to this trend; *CanDrivR-COAD (Colorectal Cancer)* only achieved an F1 score of 69%, despite its dataset size exceeding 3000 variants. Overall, our results underscore a significant gain in prediction accuracy when considering the cancer-specific context in which variants occur.

#### 3.2.2 Feature importance

We next investigated the most important features for distinguishing rare and recurrent variants. We compared the top five features for the top-performing cancer-specific models (leading to a comparison of 32 features across ten cancers). We present the results as a heatmap in Fig. 2.

The top features included DNA shape properties and conservation scores (Chiu *et al.* 2016). Despite the helix twist (HelT), propeller twist (ProT), electrostatic potential (EP), and minor groove width (MGW) DNA shape features being informative for most cancer types, their relative importance varied by sequence position. For instance, in Skin Cutaneous Melanoma (SKCM), the ProT value is most important at the site of substitution in the wild-type sequence (denoted by the value ‘11’), whereas, for UCEC, the importance of ProT is more pronounced at position 18 in the mutant sequence. This variability explains why cancer-specific models are unable to generalise well to other cancer datasets (see Fig. 6, available as supplementary data at *Bioinformatics Advances* online).

We investigated DNA shape features further for the SKCA and SKCM datasets, by plotting the mean values at each position, in both wild type and mutant sequences, for both recurrent and rare variants (Fig. 3). We found that recurrent variants occurred in DNA regions that were more bent and twisted. Conversely, rare variants exist within ‘flatter’ DNA regions.

#### 3.2.3 Evaluating Top-Performing CanDrivR-CS models on unseen TCGA data

We evaluated two top-performing models, *CanDrivR-UCEC (Uterine Corpus Endometrial Carcinoma)* and *CanDrivR-SKCM (Skin Cutaneous Melanoma)*, using data from TCGA. Our results showed that *CanDrivR-CS* models generalise well to unseen TCGA datasets (Table 4). *CanDrivR-SKCM* achieved an F1 score of 91.8%. Additionally, *CanDrivR-UCEC* performed well with an F1 score of 79.0% for the TCGA data. Hence, our cancer-specific predictors are also able to generalise to new data with far higher accuracy than our baseline pan-cancer model.

In Section 2.5, available as supplementary data at *Bioinformatics Advances* online, we further discuss classifier performance, with reference to the fact that stated classifier performance is dataset size dependent. We also consider prospective test accuracies if more data becomes available per cancer type.

## 4 Discussion and conclusion

In this study, we present *CanDrivR-CS*, a cancer-specific gradient boosting approach designed to distinguish between rare and recurrent missense variants. The primary objective of this work was to investigate whether building cancer-specific machine learning models enhances prediction accuracy, compared to a pan-cancer approach. Our secondary objective was to identify which features are important for distinguishing between rare and recurrent single nucleotide missense variants in cancer genomes. Given the heterogeneity of cancers and their tendency to exhibit a wide variety of rare somatic variants in their genomes, investigating the molecular underpinnings of these events may shed light on why some variants occur more frequently than others.

Our pan-cancer baseline model performed strongly when evaluated using the ICGC dataset, achieving a leave-one-group-out cross-validation (LOGO-CV) F1 score of 79.2%. However, when applied to unseen data from the COSMIC database, performance dropped to an F1 score of 65.6%. This discrepancy is largely attributable to how variant recurrence is defined and distributed across the datasets. In both cases, we define ‘rare’ variants as those observed in only one sample (R = 1), and ‘common’ variants as those observed in more than two samples (R > 2). However, because COSMIC is nearly twice the size of ICGC and aggregates data from broader and more heterogeneous sources, variants considered rare in ICGC may appear more frequently in COSMIC, effectively shifting them into the ‘common’ class by our definition. As a result, the model, which was trained to identify certain variants as rare based on their frequency in ICGC, may misclassify those same variants as rare in COSMIC even though they are recurrent in that dataset.

This highlights a challenge in recurrence-based labelling, especially when training and testing occur across datasets of different scale or coverage. Unlike functional annotations, which may remain relatively stable across datasets, recurrence is a dynamic property—sensitive to sample size, cohort composition, and data aggregation depth. For example, COSMIC incorporates data from multiple studies and cancer types, some of which may not be represented in ICGC, resulting in variants with artificially inflated recurrence counts. This introduces label noise when transferring models between datasets: the label of a variant can change simply due to differences in the coverage of the dataset, not due to any intrinsic biological difference.

To mitigate this, future work might explore more robust or normalised definitions of recurrence, such as adjusting for cohort size, tumour type stratification, or weighting recurrence by study origin. Alternatively, strategies like calibrating the model to the target dataset’s recurrence distribution, or using semi-supervised approaches to adjust decision boundaries post-transfer, could help improve generalisability. These steps may be especially important in variant prioritisation tasks that rely on frequency-based filtering but seek to generalise across large, heterogeneous cancer variant resources.

In the next part of our analysis, we compared the performance of our pan-cancer baseline model with cancer-specific models. Our findings indicated that developing predictors tailored to specific cancer types significantly improves model performance, compared to models trained on variants across all cancer types. While our baseline model achieved a maximum LOGO-CV F1 score of 79.2%, the *CanDrivR-READ (Rectal Adenocarcinoma)* and *CanDrivR-SKCM* models achieved cross-validation scores of 88% and 90%, respectively, representing an improvement of up to 11%. This improvement underscores the importance of tailoring models to specific cancer types.

Although larger datasets generally led to better performance, our results also highlight that factors such as data set heterogeneity, mutation burden, and cancer-specific genomic characteristics may influence model outcomes. For example, several types of cancer performed noticeably below the baseline. Specifically, *CanDrivR-LIAD (Liver Adenocarcinoma)* and *PEME (Paediatric Medulloblastoma)* reached F1 scores of only 56% and 64%, respectively. This underperformance is likely due, in part, to the limited number of labelled variants available for training (fewer than 250 in each case), which restricts the model’s ability to learn robust patterns.

However, data quantity alone does not fully account for variability. For example, *CanDrivR-MALY (Malignant Lymphoma)*, although it comprises 990 variants, achieved a comparatively modest F1 score of 70%. A plausible explanation is the high degree of *genetic heterogeneity* associated with lymphomas. MALY encompasses multiple subtypes-including diffuse large B-cell lymphoma (DLBCL), follicular lymphoma, and Hodgkin lymphoma-each with distinct mutational landscapes and driver profiles (Swerdlow *et al.* 2016). This biological diversity may dilute the signal consistency and hinder the model’s ability to generalise across the subtype spectrum. Furthermore, certain lymphomas exhibit *hypermutation* or elevated background mutation rates, which can obscure meaningful recurrence signals and increase the prevalence of passenger mutations (Pasqualucci and Dalla-Favera 2018).

Furthermore, the *informative value of the features* -such as the local sequence context or conservation-may differ depending on the dominant mutational processes in that cancer. For example, cancers driven by environmentally induced mutations (e.g. UV in skin, tobacco in lung) may follow more predictable mutational patterns, while those shaped by complex chromosomal rearrangements or epigenetic changes may be less amenable to recurrence-based classification alone (Martincorena and Campbell 2015, Alexandrov *et al.* 2016).

Finally, *data curation quality* and *noise* may vary across datasets. Cancers with lower-quality variant annotations or more heterogeneous sequencing protocols could introduce inconsistencies that reduce model performance. This is particularly relevant in rare or paediatric cancers, where sequencing depth, sample diversity, and annotation completeness may be more variable.

In this study, we opted not to perform exhaustive hyperparameter tuning for our XGBoost models, prioritising a consistent, interpretable, and reproducible modelling framework across all cancer types. This decision also simplified comparisons across cancer types and supported the scalability of our approach. However, we acknowledge that future work could explore more systematic hyperparameter optimisation, particularly for cancers with limited variant counts, where additional tuning may help improve model generalisability.

In summary, while sample size plays a clear role, our findings suggest that *cancer-type-specific biology, mutation burden, class distribution, and dataset quality* are equally critical in shaping model performance. Understanding and modelling these factors will be essential for future improvements in pan-cancer variant classification.

In the next part of our work, we investigated feature importance. Interestingly, DNA shape properties such as electrostatic potential, propeller twist, helix twist, and roll consistently ranked among the top features across most cancer types, despite our models being trained on missense variants. Specifically, we found that recurrent variants were typically located in more complex DNA regions, characterised by bends and twists. In contrast, rare variants were more likely to occur in simpler, ’flatter’ regions. We hypothesise that complex DNA shapes may be more prone to errors during the replication process, potentially acting as mutational hotspots, compared to flatter regions that are easier to replicate. To our knowledge, this is the first study to characterise the prevalence of cancer variants in these types of DNA regions. However, one previous study has shown that G-quadruplex DNA structures are correlated with SCNA breakpoint hotspots (De and Michor 2011). We speculate that regions of DNA that are more highly bent or twisted may act as mutational hotspots in both disease and healthy genomes. However, in cancer cells, due to rapid cell division and increased stress on DNA replication and repair mechanisms, these regions may evade detection and repair more easily than in normal tissues.

In this study, we developed models to classify somatic cancer variants as rare or recurrent based on observed sample-level recurrence within individual tumour types. While this provided a practical and tractable labelling strategy for studying tumour-specific variation, we emphasise that such recurrence labels do not directly correspond to biological function or clinical relevance. Variant recurrence is highly sensitive to dataset size and cohort composition—a variant labelled as rare in one cohort (e.g. ICGC) may be recurrent in another (e.g. COSMIC), and recurrence itself does not necessarily indicate a variant’s functional role in cancer.

We considered several alternative labeling approaches, including training models to predict pathogenic versus benign labels derived from sources such as ClinVar, COSMIC, or gnomAD (Landrum *et al.* 2018, Tate *et al.* 2019, Karczewski *et al.* 2020). However, these strategies introduce additional complications. First, many of these resources combine germline and somatic annotations, which can confound cancer-specific modelling. Second, and more critically, the pathogenicity labels in databases such as ClinVar are often partially derived from computational predictions (Richards *et al.* 2015). As such, using them as training labels can introduce circularity, where new models are trained to replicate decisions made by older tools, rather than learning from independent biological evidence.

In light of these limitations, we opted for a recurrence-based framework that allowed us to compare somatic variants within consistent tumour contexts, leveraging only observed frequencies. While this does not fully resolve the functional relevance of variants, it provides a reproducible basis for exploring cancer-specific modelling and feature utility. We view this study as a proof of concept, and anticipate that future work could extend this framework using higher-quality, biologically validated labels. In particular, the increasing availability of high-throughput multiplexed assays of variant effect (MAVE) may enable better resolution of pathogenic and neutral variants in tumour-specific settings, supporting more functionally interpretable models in the future.

Our first papers on predicting the pathogenic status of variants in the human genome (*FATHMM* (Shihab *et al.* 2013) and *FATHMM-MKL* (Shihab *et al.* 2015)) exploited sequence conservation measures across species. However, benign single nucleotide polymorphisms frequently congregate in less conserved regions, which might present a prediction bias. We therefore investigated a method, *CScape-somatic* (Rogers *et al.* 2020), which only used cancer-sample-derived somatic variants, with a similar distribution by class, according to sequence conservation measures. As in the current paper, this paper compared the class of rare (*n* = 1) somatic variants, with somatic variants recurrently observed in cancer samples. In this paper (Rogers *et al.* 2020) we cautioned that a rare variant (occurs once, *n* = 1 in the COSMIC archive) can be a rare driver. Recurrently observed variants in cancer samples, absent from healthy subjects, can actually be benign variants: for example, the variant might be in linkage disequilibrium with an actual driver. However, in the *CScape-somatic* paper we suggested that *n* = 1 variants are likely enriched for benign, and recurrently observed in cancer samples are likely enriched for drivers. Relative to *CScape-somatic*, the current paper therefore illustrates the significant test accuracy gains which can be made through cancer-type-specific prediction, and through the use of our *DrivR-base* (Francis *et al.* 2024) resource. However, as stated in the current paper, we note that the highest test accuracy, at 90% on unseen test data, is for melanoma (SKCM) and SKCA at 89%, frequently caused by UV-damage. Also, the weighted primary sources of data used to achieve this level of prediction accuracy are associated with the physical stability of the DNA. As commented already, this means they could be mutational hotspots, weak points in the DNA which are more easily mutated, but which are benign. For this reason, we have not associated predictions with driver-status. The currently proposed classifiers therefore only predict that a single nucleotide variant will be recurrently observed in cancer samples.

The research project outlined in this paper can be extended in multiple directions. For example, it may be possible to improve predictive accuracies using data augmentation methods (Mumuni and Mumuni 2022). Also, some cancer types have extensive commonalities, such as colon and colorectal cancer, and others can be more divergent. This allows for the possibility of weighted transfer learning across cancer types. In related contexts, using weighted transfer learning, and each cancer type a *task*, prediction accuracies have been improved through the use of *multi-task* learning methods from machine learning (Collier *et al.* 2019). This approach would therefore interpolate between our pan-cancer predictor and the cancer-type-specific predictors. As previously commented, we also did not perform an exhaustive hyperparameter tuning for our XGBoost models. Tuning might improve test accuracies a little but at the possible risk of overfitting if not pursued carefully: nevertheless this is a further avenue for later investigation. As commented earlier, a further project would be to improve generalisability across datasets by reformulating the task using relative abundance, rather than absolute sample counts for variants of interest. Finally, in previous studies (Darbyshire *et al.* 2019, Rogers *et al.* 2021), we applied these computational tools to cancer genome data across different cancer types using a previous method (Rogers *et al.* 2017). This gave interesting insights. This could be another project for exploration using the currently proposed prediction tools.

## Supplementary Material

vbag008_Supplementary_Data
