# Supplementary material for: CanDrivR-CS: a cancer-specific machine learning framework for distinguishing recurrent and rare variants
Source: Bioinform Adv. 2026 Jan 12;6(1):vbag008. doi: 10.1093/bioadv/vbag008 (PMC12935160; doi:10.1093/bioadv/vbag008)
Supplement: vbag008_Supplementary_Data [file vbag008_supplementary_data.zip › Supplementary.pdf]

Supplementary Materials for *CanDrivR-CS*: A Cancer-Specific Machine Learning Framework for Distinguishing Recurrent and Rare Variants

Amy Francis, Colin Campbell, Tom Gaunt

January 8, 2026

In this Supplementary, we present details of the software used (Section 1) and comments on our methods and models for *CanDrivR* and *CanDrivR-CS* (Section 2).

1 Software Availability and Usage Advisory

The *CanDrivR-CS* code, training, and test data is available at:

`https://github.com/amyfrancis97/CanDrivR-CS`.

This Github webpage gives the code-base which was used for determining the results in the paper. The Anaconda environment setup can fail because the environment specification pins exact builds (e.g. $= py38hecd8cb5\_0$) for Intel processors. Some of these are also defaults for Python 3.8. The code base was independently evaluated by a set of potential users, and by all authors (to also cover the possibility of using an Arm64 Mac based machine). The Anaconda environment setup failed with the Arm64 Mac for the above reasons, but evaluations on Intel processor machines proved successful: for Intel processors, modules were sometimes reported absent by some users, though with such problems readily resolved via updates. The numbers output from the code can differ marginally from the results stated in the paper, due to these module dependencies. To improve ease of usage, the authors intend to create a stand-alone Python-based software package in due course, which would be presented on this Github page. However, this would be conditional on time resource availability for this research project.

2 Methods & Optimisation

2.1 Optimising the Recurrent Dataset Threshold

2.1.1 *CanDrivR* Baseline Model

Our baseline model classified variants based on whether they are rare or recurrent in the International Cancer Genome Consortium (ICGC) dataset [4]. Rare variants were defined as those occurring only once in the ICGC dataset. To optimise the threshold for selecting recurrent variants, we altered our training data to include recurrent variants with donor counts in the range of >1 and >9. Figure 1 shows the mean cross-validation F1 score against the donor count threshold for the recurrent class. For each threshold, we randomly sub-sampled the same number of rare variants ($r = 1$) to create a balanced dataset. We found that a donor count threshold of >2 led

to the highest F1 score (approximately 78%), while still maintaining a dataset size of 135,648 missense variants. As a result, we adopted this threshold in the *CanDrivR* baseline model.

2.1.2 *CanDrivR-CS* Models

We then repeated this threshold optimisation process for each of our *CanDrivR-CS* models, adjusting the threshold for each cancer type (Figure 2). For each cancer type, we selected the threshold that resulted in the highest performance.

2.2 Feature Selection

We performed feature selection to identify the most informative features for distinguishing between rare and recurrent mutations. Initially, our baseline XGBoost model used 1,807 features derived from *DrivR-Base* [2]. For optimisation purposes, we randomly sampled a balanced training subset of 5,000 variants from the baseline ICGC dataset. The importance of each feature was evaluated using XGBoost's get_booster().get_score function, which ranks features based on their contribution to the model.
In the initial run, 673 out of the 1,807 features were found to be informative; each receiving an importance score of 1 or higher. These 673 features were then ranked by importance, with the top feature being hg48phyloP470way (importance score = 37). To assess how feature inclusion impacted the performance, we employed an iterative approach. Starting with the most important feature, we incrementally added features to the model, performing 673 iterations in total. For each iteration, we plotted the number of features used against the mean F1 score obtained through leave-one-group-out cross-validation (blue line). The standard deviation of the F1 scores across the folds is represented by the orange line. From this, we decided to set a threshold of 351 features for the final model, as including additional features beyond this point did not improve the performance (Figure 3).
From the selected features, we plotted the top 30 based on their importance scores in XGBoost (Figure 4). The most informative features were conservation-based metrics such as PhyloP and PhastCons, alongside DNA-shape properties predicted by DNAShapeR. These DNA-shape properties included Rolls, Helix Twists (HelT), and Minor Groove Width (MGW).

2.3 *CanDrivR-CS*: How Well Can Cancer-Specific Models Generalise to Other Cancer Datasets?

We aimed to understand the extent to which our cancer-specific models could generalise to other cancer datasets. The rationale behind this testing was for two reasons: firstly, to investigate whether certain cancer types can be pooled into a single predictive model, and secondly, to explore the potential for shared feature group importances across different cancers. By training models on specific cancer types and then testing them on other cancer datasets, we recorded the test accuracy and the F1 score for all models on each dataset. The results for the top models are shown in Supplementary Figure 5, where the orange point indicates the test results for the cancer dataset used to train the model.
These test results demonstrated that cancer-specific models were able to generalise to other cancer types, but typically with a much lower performance, compared to a cancer-specific context. Across all models, no other datasets showed competing performance with the cancer that they were trained on. This suggests that there is an advantage in building cancer-specific predictors, as each cancer has unique variants that exist within rare and recurrent backgrounds.

2.4 Final Features

In Tables 2 to 7, we list all of the features used in our final model with a brief description and their sources. For a full explanation and description of the feature groups used in *CanDrivR-CS*, please refer to our previous *DrivR-Base* paper and supplementary materials [2].

2.5 Test Set Accuracy Dependence on Sample Size

A comparison of the test set accuracies of cancer-type predictors in Table 1 of the main paper is made complex by the dependence of test accuracy on training set size. Within the machine learning literature, there is theoretical and experimental support for a Zipf's law dependence of the *test error*, z, on the training set size, m. That is, $z = am^{-\alpha} + b$. The parameter b is a residual error which might, for example, derive from unstructured white noise in the data, or there may be a lack of key input data which negates perfect classification, even in the theoretical case of $m \to \infty$. The parameter α is related to the efficiency of the learning algorithm used to build the classifier, with an upper bound of $\alpha = 1$ for the most efficient algorithms [7]. In [5] we used such an insight to investigate the dependence of the test error of a classifier on the training set size, for gene expression data. In this paper we show that, for a given gene expression dataset, it is possible to determine a, b and α from the current dataset, and hence extrapolate classifier performance if more data were to become available (the $m \to \infty$ limit). Our co-authored paper [5] has citations to various machine learning papers relevant to this discussion of learning curves. In Supplementary Figure 6 we present the *test set accuracies* (TSA, i.e. $1 - z$) for the 50 cancer types considered in this study, stated in Section 2.6 below, and described in the main paper, Table 2. We derived a minimally placed Zipf's Law which passes through the weakest classifiers in terms of test performance (rendered as a test accuracy curve in Supplementary Figure 6), against training set size. All depicted test accuracies lie on or above this lower bounding learning curve. Though only an estimation process, this approach does give interesting insights. Firstly, for 20 of the 50 cancer types considered, the *test set accuracies* are on or above 0.8. This lower bound on the most accurate classifiers is presented as a dashed horizontal line in Supplementary Figure 6. Secondly, for poorer test set accuracy prediction, such as liver adenocarcinoma (LIAD) at 0.6 and uterine carcinosarcoma (UTCA) at 0.63, these test set accuracies are most probably due to inadequate training set sizes. Improvements can be made with more data. Thirdly, this argument also suggests that, if a sufficient number of samples become available, then there is a minimal test set accuracy of about 0.7 achievable for all classifiers i.e. *taken across all cancer types.* Finally, this approach also suggests that, due to heterogeneity in the data, or poor characterisation of functionally distinct subtypes, perfect classification is generally unattainable even in the theoretical limit of $m \to \infty$. Noise in the data could contribute to this shortfall in the TSA as $m \to \infty$. However, such experimental noise is likewise present for the 20 classifiers with TSAs on or above 0.8, so this is not a complete explanation. The most likely explanations, related to the b parameter in Zipf's Law, is that the classifier lacks crucial input information which is required for a higher accuracy prediction, that the cancer genetic profile is very heterogeneous, or that the phenotype descriptions are inadequate.

2.6 Data behind Figure 1 of the main paper and Supplementary Figure 6

With reference to the cancer names and corresponding type-codes of Table 2 of the main paper (cf. caption comment on LAML and CMDI), the table below restates the numbers behind Figure 1 of the main paper in alphabetic order:

Cancer type	Test set accuracy (TSA)	Prec	Rec	F1	AUC
ALL	0.63	0.66	0.69	0.65	0.73
AML	0.57	0.55	0.70	0.60	0.58
BLCA	0.78	0.78	0.79	0.78	0.84
BOCA	0.73	0.69	0.74	0.70	0.78
BRCA	0.78	0.76	0.81	0.78	0.85
BTCA	0.79	0.79	0.79	0.79	0.84
CESC	0.83	0.83	0.84	0.83	0.90
CLLE	0.70	0.71	0.76	0.72	0.71
CMDI	0.70	0.71	0.68	0.68	0.79
COAD	0.70	0.71	0.68	0.69	0.75
COCA	0.76	0.76	0.76	0.76	0.82
DLBC	0.65	0.71	0.65	0.67	0.66
EOPC	0.72	0.74	0.69	0.71	0.79
ESAD	0.79	0.79	0.80	0.79	0.85
ESCA	0.79	0.81	0.79	0.80	0.85
GACA	0.82	0.82	0.83	0.82	0.89
GBM	0.84	0.85	0.84	0.84	0.91
HNSC	0.84	0.84	0.84	0.84	0.89
KICH	0.72	0.73	0.68	0.70	0.77
KIRC	0.66	0.67	0.67	0.66	0.73
KIRP	0.73	0.73	0.75	0.74	0.79
LAML	0.68	0.67	0.70	0.68	0.72
LGG	0.85	0.91	0.80	0.84	0.91
LIAD	0.60	0.58	0.57	0.56	0.64
LICA	0.85	0.84	0.88	0.86	0.91
LIHC	0.82	0.80	0.86	0.83	0.88
LINC	0.78	0.74	0.85	0.79	0.82
LIRI	0.59	0.58	0.49	0.52	0.65
LMS	0.78	0.78	0.80	0.78	0.82
LUAD	0.82	0.80	0.89	0.84	0.89
LUSC	0.82	0.81	0.86	0.83	0.88
MALY	0.65	0.65	0.77	0.70	0.63
MELA	0.71	0.77	0.69	0.70	0.76
NACA	0.73	0.72	0.72	0.72	0.77
NKTL	0.64	0.64	0.67	0.65	0.67
ORCA	0.84	0.82	0.85	0.83	0.91
OV	0.83	0.83	0.84	0.83	0.88
PAAD	0.84	0.85	0.83	0.84	0.90
PACA	0.83	0.85	0.79	0.82	0.88
PAEN	0.69	0.70	0.72	0.66	0.80
PBCA	0.79	0.77	0.78	0.77	0.85
PEME	0.63	0.67	0.65	0.64	0.69
PRAD	0.82	0.83	0.81	0.82	0.87
READ	0.89	0.86	0.90	0.88	0.95
RECA	0.66	0.68	0.69	0.67	0.72
SARC	0.66	0.66	0.67	0.66	0.73
SKCA	0.89	0.89	0.89	0.89	0.92
SKCM	0.90	0.90	0.91	0.90	0.96
STAD	0.84	0.85	0.82	0.84	0.91
THCA	0.84	0.83	0.87	0.85	0.90
UCEC	0.88	0.89	0.87	0.88	0.94
UTCA	0.63	0.61	0.63	0.61	0.67

References

[1] Tsu Pei Chiu, Federico Comoglio, Tianyin Zhou, Lin Yang, Renato Paro, and Remo Rohs. DNAshapeR: an R and BioConductor package for DNA shape prediction and feature encoding. *Bioinformatics*, 32:1211, 4 2016.

[2] Amy Francis, Colin Campbell, and Tom R. Gaunt. DrivR-Base: a feature extraction toolkit for variant effect prediction model construction. *Bioinformatics*, 40, 3 2024.

[3] Maik Friedel, Swetlana Nikolajewa, Jürgen Sühnel, and Thomas Wilhelm. DiProDB: a database for dinucleotide properties. *Nucleic Acids Research*, 37:D37, 2009.

[4] Thomas J. Hudson, Warwick Anderson, Axel Artez, and Anna D Barker*et al.* International network of cancer genome projects. *Nature*, 464:993, 4 2010.

[5] Sayan Mukherjee, Pablo Tamayo, Simon Rogers, Ryan Rifkin, Anna Engle, Colin Campbell, Todd Golub, and Jill Mesirov. Estimating dataset size requirements for classifying DNA microarray data. *Journal of Computational Biology*, 10:119–142, 2003.

[6] Luis R. Nassar, Galt P. Barber, Anna Benet-Pagès, and Jonathan Casper *et al.* The UCSC genome browser database: 2023 update. *Nucleic acids research*, 51:D1188–D1195, 1 2023.

[7] Manfred Opper and David Haussler. Generalization performance of Bayes optimal classification algorithms for learning a perceptron. *Phys. Rev. Letters*, 66:2677–2680, 1991.

[8] Julien Pelé, Jean Michel Bécu, Hervé Abdi, and Marie Chabbert. Bios2mds: An R package for comparing orthologous protein families by metric multidimensional scaling. *BMC Bioinformatics*, 13:1–7, 6 2012.

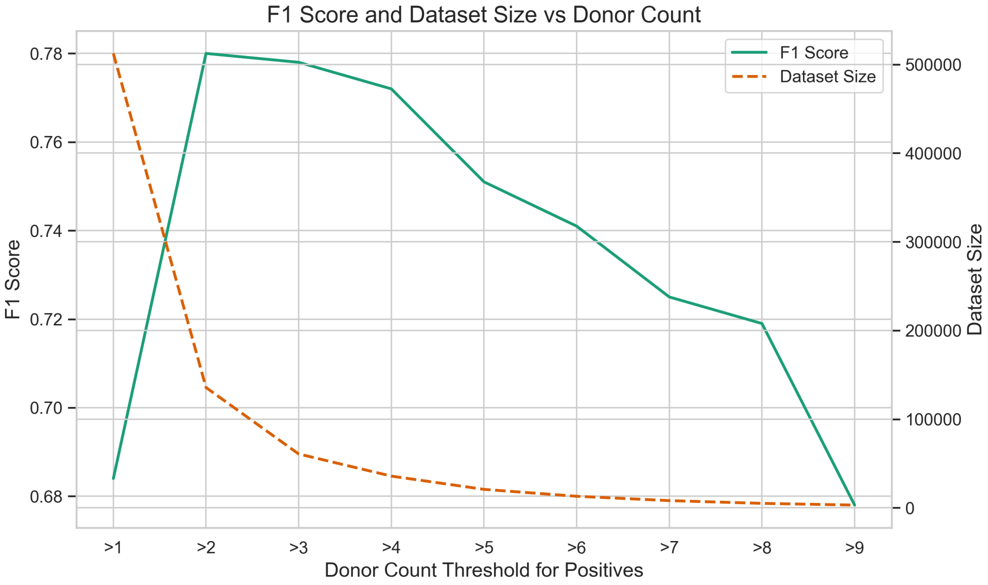

Figure 1: We aimed to establish the best threshold for filtering our recurrent variants. We trained the model using different donor count thresholds ranging from >1 to > 9, and evaluated the models on our cancer-specific test data. We found that filtering variants to those that are found in more than two patients yielded the highest F1 cross-validation score (0.78).

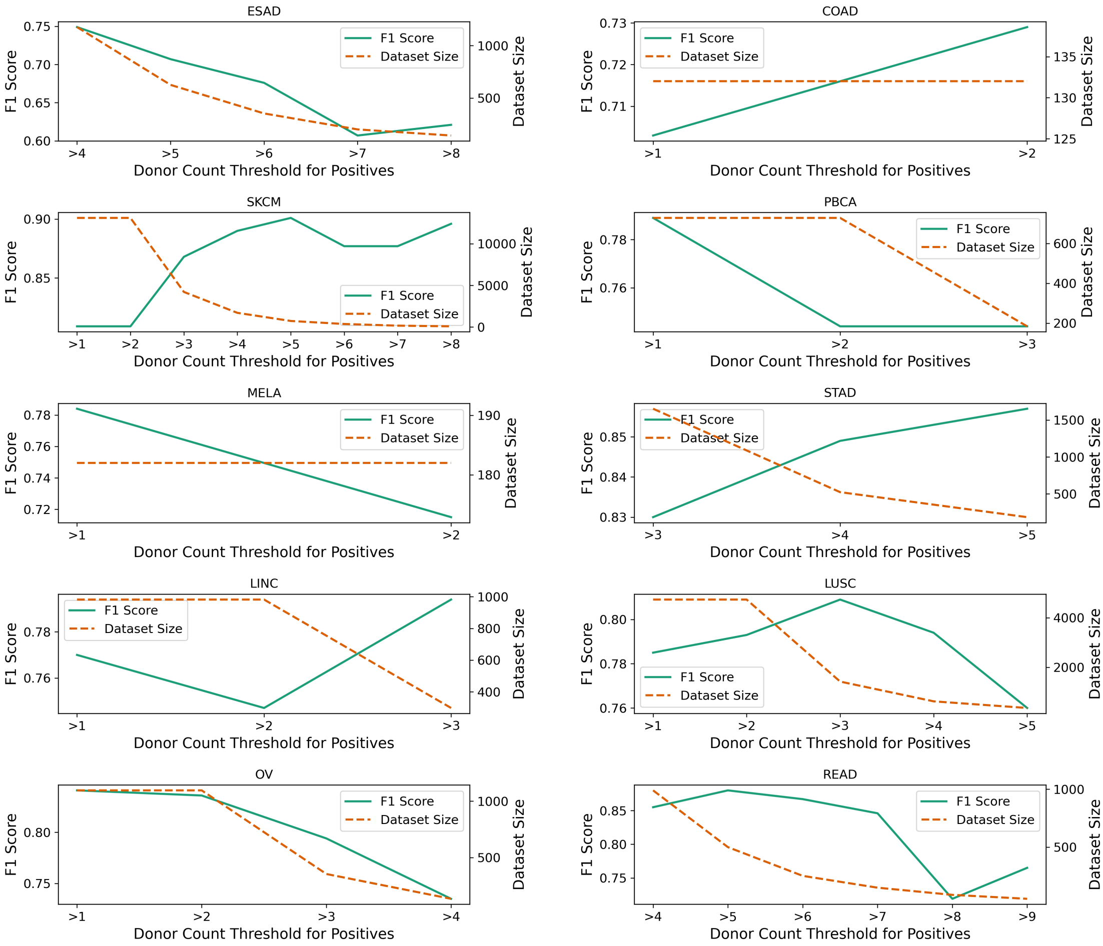

Figure 2: For each cancer type in *CanDrivR-CS*, we optimised the donor count threshold of the recurrent variants. We select the threshold that led to the highest performance for each dataset. Here we show some examples of the optimisation curves produced.

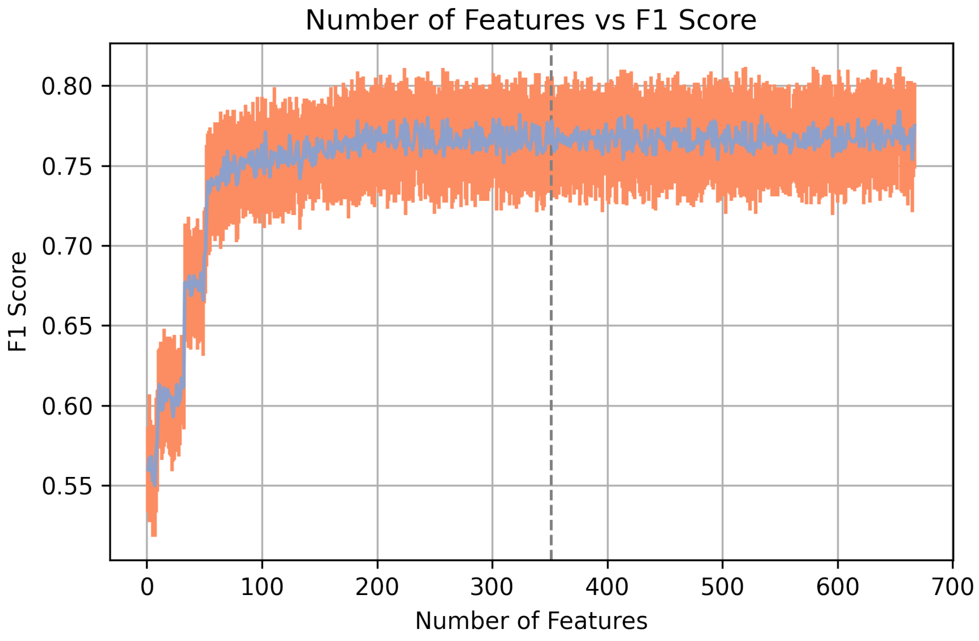

Figure 3: We optimised the features used in our model through sequential feature selection. To reduce the data size for optimisation purposes, we randomly sub-sampled our training dataset to create a balanced subset of 5,000 variants. We then employed XGBoost's get_booster().get_score function to obtain feature importance scores, ranking the top 673 features that had a score greater than 1. Starting with the most important feature, we incrementally added the next most important feature to the model and re-evaluated our baseline model. We plotted the mean cross-validation F1 score for each iteration (in blue) alongside the standard deviation across the folds (in orange). The grey line shows the selected cutoff for diminishing returns.

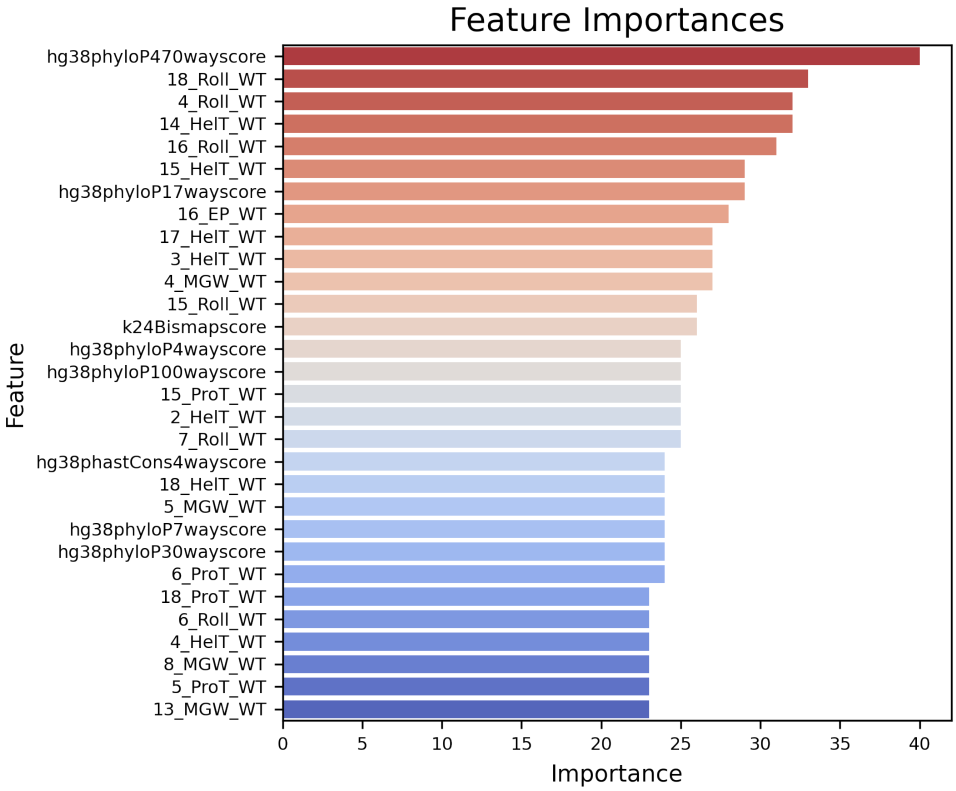

Figure 4: From our features selected in Figure 3, we plotted the top 30 features with their relative importance scores. For more information on the feature codes, please refer to supplementary tables 2-7

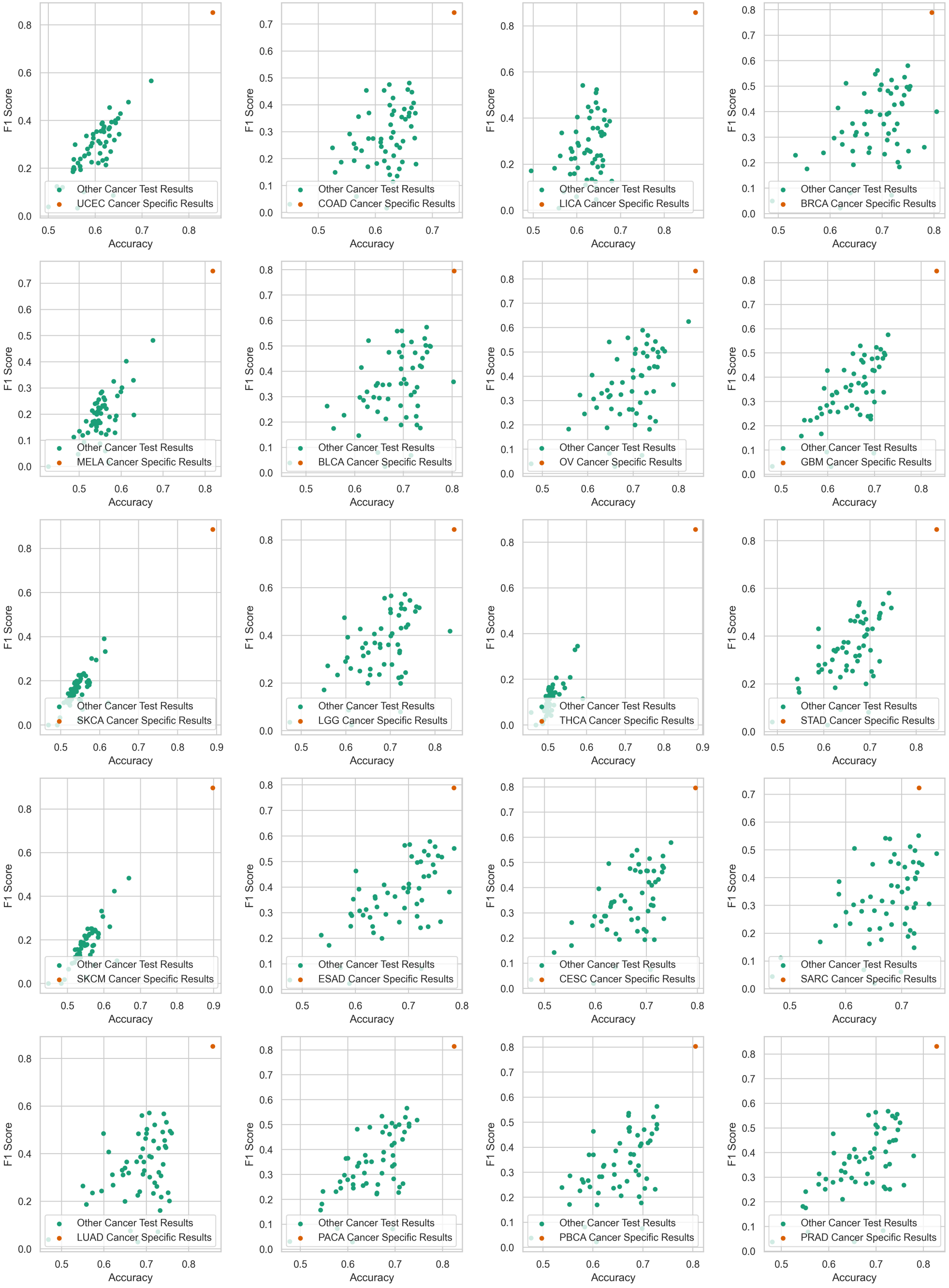

Figure 5: Here, we plotted the cancer-specific cross-validation F1 score vs accuracy against the results of testing on all other cancer datasets. Specifically, we trained our *CanDrivR-CS* models, and tested each model on all other cancer datasets. The reasoning for this was to test the ability of our models to generalise to other cancer datasets. We found that for each cancer model, the corresponding cancer test dataset gives the best performance. No other cancer datasets are competitive or even close to the performance of the cancer data that was used to train the model. Hence, we cannot pool any cancer-specific models together.

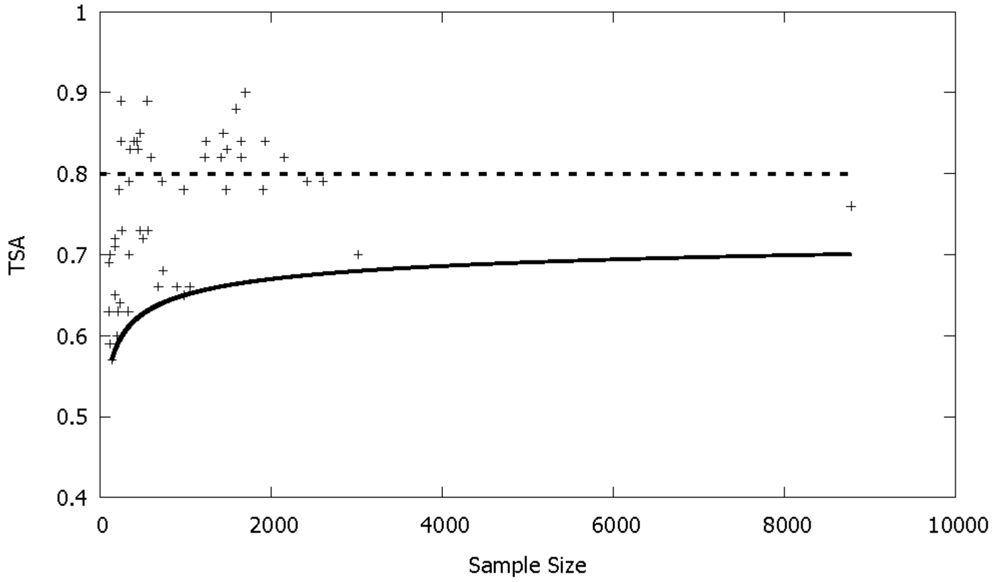

Figure 6: For the 50 cancer types considered in Table 1 of the main paper, this Figure gives the test set accuracy (TSA, on the y-axis) against the corresponding sample size (x-axis). For 20 of the cancer types, the TSA is lower bounded by 0.8 given as a dashed line in the Figure. All datapoints are lower bounded by a Zipf's Law curve (see text) which indicates that, for some cancer types, increasing the sample size would probably raise the TSA to 0.7 or better. The asymptotic performance of this learning curve ($m \to \infty$, m is the sample size) suggests perfect classification is unattainable due to noise in the data, crucial data is lacking, and other factors.

Table 1: Missing Data Summary

Feature Name	Missing Count	Missing %
k24Bismapscore	14430	10.64
PHAT	9607	7.08
BLOSUM45	9607	7.08
JTT_TM	9607	7.08
BLOSUM30	9607	7.08
PAM120	9607	7.08
JTT	9607	7.08
BLOSUM80	9607	7.08
PAM40	9607	7.08
PAM250	9607	7.08
mutant_AA_Average_relative_fractional _occurrence_in_E0(i)_(Rackovsky-Scheraga,_1982)	9490	7.00
mutant_AA_Normalized_positional _residue_frequency_at_helix_termini_C4'(Aurora-Rose,_1998)	9490	7.00
mutant_AA_Normalized_positional _residue_frequency_at_helix_termini_Nc_(Aurora-Rose,_1998)	9490	7.00
mutant_AA_Normalized_positional _residue_frequency_at_helix_termini_C"(Aurora-Rose,_1998)	9490	7.00
mutant_AA_Normalized_positional _residue_frequency_at_helix_termini_C'(Aurora-Rose,_1998)	9490	7.00
mutant_AA_Normalized_positional _residue_frequency_at_helix_termini_N"(Aurora-Rose,_1998)	9490	7.00
mutant_AA_Normalized_positional _residue_frequency_at_helix_termini_N'(Aurora-Rose,_1998)	9490	7.00
mutant_AA_Normalized_positional _residue_frequency_at_helix_termini_N4(Aurora-Rose,_1998)	9490	7.00
mutant_AA_Normalized_positional _residue_frequency_at_helix_termini_N3(Aurora-Rose,_1998)	9490	7.00
mutant_AA_Normalized_positional _residue_frequency_at_helix_termini_N2(Aurora-Rose,_1998)	9490	7.00
mutant_AA_Normalized_positional _residue_frequency_at_helix_termini_N1(Aurora-Rose,_1998)	9490	7.00

Continued on next page

Table 1 – continued from previous page

Feature Name	Missing Count	Missing %
mutant_AA_Normalized_positional_residue_frequency_at_helix_termini_C1(Aurora-Rose,_1998)	9490	7.00
mutant_AA_Normalized_positional_residue_frequency_at_helix_termini_C2(Aurora-Rose,_1998)	9490	7.00
mutant_AA_Normalized_positional_residue_frequency_at_helix_termini_C3(Aurora-Rose,_1998)	9490	7.00
mutant_AA_Normalized_positional_residue_frequency_at_helix_termini_C4(Aurora-Rose,_1998)	9490	7.00
mutant_AA_Average_relative_probability_of_beta-sheet_(Chou-Fasman,_1978a)	9490	7.00
mutant_AA_Average_relative_probability_of_alpha-helix_(Chou-Fasman,_1978a)	9490	7.00
mutant_AA_Hydropathy_scale_based_on_self-information_values_in_the_two-state_model_(50%_accessibility)_(Naderi-Manesh_et_al.,_2001)	9490	7.00
mutant_AA_Hydrophobicity_coefficient_in_RP-HPLC,_C18_with_0.1%TFA/2-PrOH/MeCN/H2O_(Wilce_et_al._1995)	9490	7.00
mutant_AA_Hydrophobicity_coefficient_in _RP-HPLC,_C8_with_0.1%TFA/MeCN/H2O_(Wilce_et_al._1995)	9490	7.00
mutant_AA_Hydrophobicity_coefficient_in _RP-HPLC,_C4_with_0.1%TFA/MeCN/H2O_(Wilce_et_al._1995)	9490	7.00
mutant_AA_Hydrophobicity_coefficient_in _RP-HPLC,_C2_with_0.1%TFA/MeCN/H2O_(Wilce_et_al._1995)	9490	7.00
mutant_AA_Hydrophobicity_coefficient_in _RP-HPLC,_C1_with_0.1%TFA/MeCN/H2O_(Wilce_et_al._1995)	9490	7.00
mutant_AA_A_parameter_defined_from_the_residuals_obtained_from_the_best_correlation_of_the_Chou-Fasman_parameter_of_beta-sheet_(Charton-Charton,_1983)	9490	7.00
mutant_AA_Hydrophobicity_coefficient_in _RP-HPLC,_C18_with_0.1%TFA/MeCN/H2O_(Wilce_et_al._1995)	9490	7.00
mutant_AA_Hydrophobicity_coefficient_in_RP-HPLC,_C8_with_0.1%TFA/2-PrOH/MeCN/H2O_(Wilce_et_al._1995)	9490	7.00
mutant_AA_Hydrophobicity_coefficient_in_RP-HPLC,_C4_with_0.1%TFA/2-PrOH/MeCN/H2O_(Wilce_et_al._1995)	9490	7.00

Continued on next page

Table 1 – continued from previous page

Feature Name	Missing Count	Missing %
mutant_AA_Hydrophobicity_coefficient_in _RP-HPLC,_C2_with_0.1%TFA/2-PrOH/MeCN/H2O_(Wilce_et_al._1995)	9490	7.00
mutant_AA_Hydrophobicity_coefficient_in _RP-HPLC,_C1_with_0.1%TFA/2-PrOH/MeCN/H2O_(Wilce_et_al._1995)	9490	7.00
WT_AA_Average_relative_fractional_occurrence_in _E0(i)_(Rackovsky-Scheraga,_1982)	9490	7.00
WT_AA_Normalized_positional_residue_frequency_at _helix_termini_C4'(Aurora-Rose,_1998)	9490	7.00
WT_AA_Normalized_positional_residue_frequency_at _helix_termini_Nc_(Aurora-Rose,_1998)	9490	7.00
WT_AA_Normalized_positional_residue_frequency_at _helix_termini_C"(Aurora-Rose,_1998)	9490	7.00
WT_AA_Normalized_positional_residue_frequency_at _helix_termini_C'(Aurora-Rose,_1998)	9490	7.00
WT_AA_Normalized_positional_residue_frequency_at _helix_termini_N"(Aurora-Rose,_1998)	9490	7.00
WT_AA_Normalized_positional_residue_frequency_at _helix_termini_N'(Aurora-Rose,_1998)	9490	7.00
WT_AA_Normalized_positional_residue_frequency_at _helix_termini_N4(Aurora-Rose,_1998)	9490	7.00
WT_AA_Normalized_positional_residue_frequency_at _helix_termini_N3(Aurora-Rose,_1998)	9490	7.00
WT_AA_Normalized_positional_residue_frequency_at _helix_termini_N2(Aurora-Rose,_1998)	9490	7.00
WT_AA_Normalized_positional_residue_frequency_at _helix_termini_N1(Aurora-Rose,_1998)	9490	7.00
WT_AA_Normalized_positional_residue_frequency_at _helix_termini_C1(Aurora-Rose,_1998)	9490	7.00
WT_AA_Normalized_positional_residue_frequency_at _helix_termini_C2(Aurora-Rose,_1998)	9490	7.00
WT_AA_Normalized_positional_residue_frequency_at _helix_termini_C3(Aurora-Rose,_1998)	9490	7.00
WT_AA_Normalized_positional_residue_frequency_at _helix_termini_C4(Aurora-Rose,_1998)	9490	7.00
WT_AA_Average_relative_probability_of _beta-sheet_(Chou-Fasman,_1978a)	9490	7.00
WT_AA_Average_relative_probability_of _alpha-helix_(Chou-Fasman,_1978a)	9490	7.00
WT_AA_Hydropathy_scale_based_on_self-information _values_in_the_two-state_model_(50%_accessibility)_(Naderi-Manesh_et_al.,_2001)	9490	7.00

Continued on next page

Table 1 – continued from previous page

Feature Name	Missing Count	Missing %
WT_AA_Hydrophobicity_coefficient_in _RP-HPLC,_C18_with_0.1%TFA/2-PrOH/MeCN/H2O_(Wilce_et_al._1995)	9490	7.00
WT_AA_Hydrophobicity_coefficient_in _RP-HPLC,_C8_with_0.1%TFA/MeCN/H2O_(Wilce_et_al._1995)	9490	7.00
WT_AA_Hydrophobicity_coefficient_in _RP-HPLC,_C4_with_0.1%TFA/MeCN/H2O_(Wilce_et_al._1995)	9490	7.00
WT_AA_Hydrophobicity_coefficient_in _RP-HPLC,_C2_with_0.1%TFA/MeCN/H2O_(Wilce_et_al._1995)	9490	7.00
WT_AA_Hydrophobicity_coefficient_in _RP-HPLC,_C1_with_0.1%TFA/MeCN/H2O_(Wilce_et_al._1995)	9490	7.00
WT_AA_A_parameter_defined_from_the_residuals _obtained_from_the_best_correlation_of_the_Chou-Fasman_parameter_of_beta-sheet_(Charton-Charton,_1983)	9490	7.00
WT_AA_Hydrophobicity_coefficient_in _RP-HPLC,_C18_with_0.1%TFA/MeCN/H2O_(Wilce_et_al._1995)	9490	7.00
WT_AA_Hydrophobicity_coefficient_in _RP-HPLC,_C8_with_0.1%TFA/2-PrOH/MeCN/H2O_(Wilce_et_al._1995)	9490	7.00
WT_AA_Hydrophobicity_coefficient_in _RP-HPLC,_C4_with_0.1%TFA/2-PrOH/MeCN/H2O_(Wilce_et_al._1995)	9490	7.00
WT_AA_Hydrophobicity_coefficient_in _RP-HPLC,_C2_with_0.1%TFA/2-PrOH/MeCN/H2O_(Wilce_et_al._1995)	9490	7.00
WT_AA_Hydrophobicity_coefficient_in _RP-HPLC,_C1_with_0.1%TFA/2-PrOH/MeCN/H2O_(Wilce_et_al._1995)	9490	7.00
k24Bismapscore_WT	8853	6.53
PHAT_WT	3853	2.84
BLOSUM45_WT	3853	2.84
JTT_TM_WT	3853	2.84
BLOSUM30_WT	3853	2.84
PAM120_WT	3853	2.84
JTT_WT	3853	2.84
BLOSUM80_WT	3853	2.84
PAM40_WT	3853	2.84
PAM250_WT	3853	2.84
PHAT_mutant	3853	2.84
BLOSUM45_mutant	3853	2.84
JTT_TM_mutant	3853	2.84
BLOSUM30_mutant	3853	2.84
PAM120_mutant	3853	2.84
JTT_mutant	3853	2.84

Continued on next page

Table 1 – continued from previous page

Feature Name	Missing Count	Missing %
BLOSUM80_mutant	3853	2.84
PAM40_mutant	3853	2.84
PAM250_mutant	3853	2.84
Resno	0	0.00
Seq	0	0.00
Pos	0	0.00
UniProt	0	0.00
mutant_AA	0	0.00
WT_AA	0	0.00
WT_AA_volume	0	0.00
mutant_AA_volume	0	0.00

Table 1: This table shows the missingness of data managed by XGBoost. [1].

Code	Positions	Description
**Wild Type (WT)**		
HelT_WT	2-18	Helical Twist
Roll_WT	2-18	Roll
ProT_WT	2-18	Propeller Twist
EP_WT	2-18	Electrostatic Potential
MGW_WT	2-8	Minor Groove Width
**Mutant (Mut)**		
HelT_Mut	9,-13	Helical Twist
Roll_Mut	8-13	Roll
ProT_Mut	9-13	Propeller Twist
EP_Mut	9-13	Electrostatic Potential
MGW_Mut	9-13	Minor Groove Width

Table 2: This table shows the DNA shape feature used in our final model. They include helix twists, propeller twists, electrostatic potential, and minor groove width. All of our DNA shape features were sourced from the R package 'DNAShapeR' [1].

Feature Code	Description
**PhyloP Scores**	
hg38phyloP470wayscore	PhyloP score across 470 vertebrate species
hg38phyloP17wayscore	PhyloP score across 17 vertebrate species
hg38phyloP7wayscore	PhyloP score across 7 vertebrate species
hg38phyloP100wayscore	PhyloP score across 100 vertebrate species
hg38phyloP4wayscore	PhyloP score across 4 vertebrate species
hg38phyloP30wayscore	PhyloP score across 30 vertebrate species
hg38phyloP20wayscore	PhyloP score across 20 vertebrate species
**PhastCons Scores**	
hg38phastCons4wayscore	PhastCons score across 4 vertebrate species
hg38phastCons17wayscore	PhastCons score across 17 vertebrate species
hg38phastCons30wayscore	PhastCons score across 30 vertebrate species
hg38phastCons20wayscore	PhastCons score across 20 vertebrate species
hg38phastCons470wayscore	PhastCons score across 470 vertebrate species
hg38phastCons7wayscore	PhastCons score across 7 vertebrate species
hg38phastCons100wayscore	PhastCons score across 100 vertebrate species
**Mappability Scores**	
k24Bismapscore	Bismap score (24-mer)
k36Bismapscore	Bismap score (36-mer)
k100Bismapscore	Bismap score (100-mer)
k100Umapscore	Umap score (100-mer)
k50Umapscore	Umap score (50-mer)

Table 3: These features represent the conservation-based and mappability scores used in our final models. We downloaded conservation measures from the UCSC Genome Browser [6].

Feature Code	Description
**GC Content**	
$< n >$GCContent	where 'n' is the GC content calculated over 20, 40, 60, 80, 100, 200, 500, 1000, 2000 base pair windows
**CpG Count**	
$< n >$CpGCount	where 'n' is the CpG count calculated over 20, 60, 100, 200, 500, 1000, 2000 base pair windows
**Observed/Expected CpG Ratio (CpG_obs_exp)**	
$< n >$CpG_obs_exp	Observed/expected CpG ratio, where 'n' is 20, 40, 60, 80, 100, 200, 500, 1000, 2000 base pair windows

Table 4: This table presents the GC content, CpG count, and observed vs expected CpG ratio for window sizes ranging between 20-2000 base pairs. For a full description of these features and how they were calculated, please refer to our *DrivR-Base* paper [2].

Feature Code	Description
10_3_x	Spectrum feature for a window size of 10 and k-mer size of 3
10_2_x	Spectrum feature for a window size of 10 and k-mer size of 2
4_2_x	Spectrum feature for a window size of 4 and k-mer size of 2
10_1_x	Spectrum feature for a window size of 10 and k-mer size of 1
8_2_x	Spectrum feature for a window size of 8 and k-mer size of 2
6_1_x	Spectrum feature for a window size of 6 and k-mer size of 1
8_1_x	Spectrum feature for a window size of 8 and k-mer size of 1
2_1_w	Spectrum feature for a window size of 2 and k-mer size of 1
10_2_z	Spectrum feature for a window size of 10 and k-mer size of 2
8_1_z	Spectrum feature for a window size of 8 and k-mer size of 1
10_1_z	Spectrum feature for a window size of 10 and k-mer size of 1
6_1_z	Spectrum feature for a window size of 6 and k-mer size of 1
8_2_z	Spectrum feature for a window size of 8 and k-mer size of 2
6_2_z	Spectrum feature for a window size of 6 and k-mer size of 2
4_1_z	Spectrum feature for a window size of 4 and k-mer size of 1
10_3_z	Spectrum feature for a window size of 10 and k-mer size of 3
6_3_z	Spectrum feature for a window size of 6 and k-mer size of 3
6_5_z	Spectrum feature for a window size of 6 and k-mer size of 5

Table 5: Feature codes and descriptions for kernel-based spectrum features. The features were derived using k-mer and window size combinations from wild type and mutant sequences. Please see our *DrivR-Base* paper for a detailed description of the calculations [2]

Feature Code	Description
JTT	Jones-Taylor-Thornton (JTT) substitution matrix
PAM40	Point Accepted Mutation (PAM) matrix, 40% divergence
GONNET	Gonnet substitution matrix
BLOSUM30	BLOSUM (BLOcks Substitution Matrix) for sequences with 30% identity
BLOSUM80	BLOSUM (BLOcks Substitution Matrix) for sequences with 80% identity
BLOSUM45	BLOSUM (BLOcks Substitution Matrix) for sequences with 45% identity
JTT_TM	Jones-Taylor-Thornton (JTT) matrix for transmembrane proteins
PHAT	PHAT (Percent Hit Acceptance Threshold) substitution matrix

Table 6: Feature codes and descriptions for substitution matrices. We show the substitution matrices that were used as features in our final model, all of which where taken from the Bio2mds package in R [8].

Feature Code	Description
Inclination	Positions: left and right of the variant (WT and Mutant)
Direction	Positions: left and right of the variant (WT and Mutant)
Probability_contacting_nucleosome_core	Positions: left and right of the variant (WT)
Stacking_energy	Positions: left and right of the variant (WT, RNA)
Twist_tilt	Positions: left and right of the variant (WT)
Twist_shift	Positions: left and right of the variant (WT and Mutant)
Tilt_roll	Positions: left and right of the variant (Mutant)
Flexibility_shift	Positions: left and right of the variant (WT)
Hydrophilicity_(RNA)	Positions: left and right of the variant (Mutant)
Minor_Groove_Size	Positions: left and right of the variant (WT and Mutant)
Entropy_(RNA)	Positions: left and right of the variant (WT)
Tilt_(RNA)	Positions: left and right of the variant (WT and Mutant)
Bend	Positions: left and right of the variant (WT and Mutant)
Shift_shift	Positions: right of the variant (WT)
Twist_twist	Positions: right of the variant (Mutant)
Shift_slide	Positions: right of the variant (Mutant)
Minor_Groove_Depth	Positions: right of the variant (Mutant)
Propeller_Twist	Positions: left of the variant (Mutant)
Tilt_(DNA-protein_complex)	Positions: left of the variant (Mutant)
Tip	Positions: left of the variant (Mutant)
Guanine_content	Positions: right of the variant (WT)
Thymine_content	Positions: right of the variant (WT)
Major_Groove_Distance	Positions: right of the variant (WT)
Slide_rise	Positions: right of the variant (WT)
Wedge	Positions: left of the variant (WT)
Shift_(DNA-protein_complex)	Positions: left of the variant (WT)
Slide_(RNA)	Positions: left of the variant (WT)
Tilt_roll	Positions: left of the variant (WT)
Twist_slide	Positions: left of the variant (WT)
Major_Groove_Width	Positions: left of the variant (WT)

Table 7: Feature codes and descriptions for dinucleotide properties from DiProDB [3]. For a detailed description on the position of the feature, please refer to *DrivR-Base* supplementary material [2].
