# Supplementary figures and images for: CanDrivR-CS: a cancer-specific machine learning framework for distinguishing recurrent and rare variants
Source: Bioinform Adv. 2026 Jan 12;6(1):vbag008. doi: 10.1093/bioadv/vbag008 (PMC12935160; doi:10.1093/bioadv/vbag008)

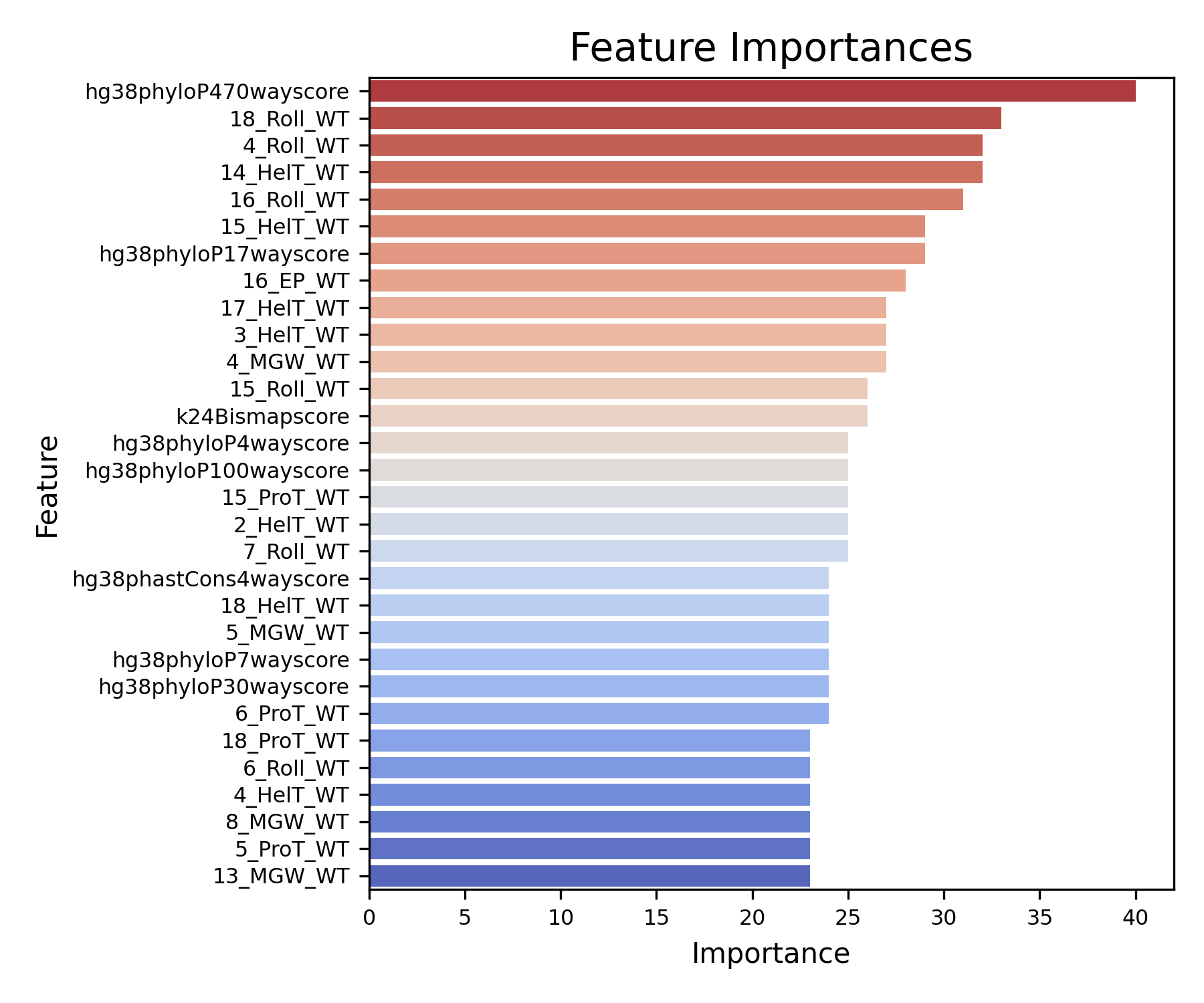

Supplement: vbag008_Supplementary_Data [file vbag008_supplementary_data.zip › figures/feature_importance.png]

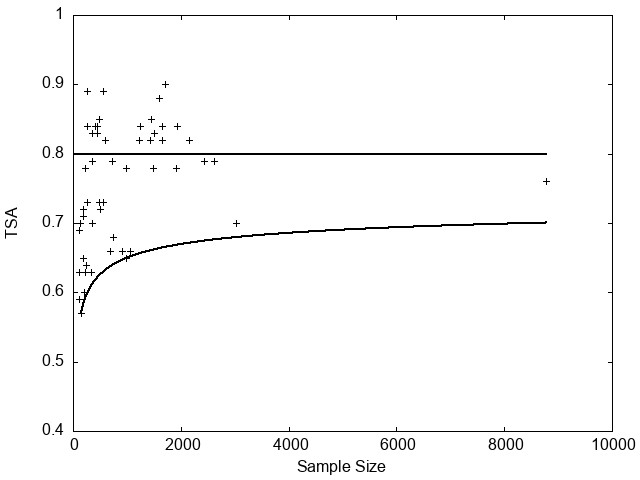

Supplement: vbag008_Supplementary_Data [file vbag008_supplementary_data.zip › acc.jpg]

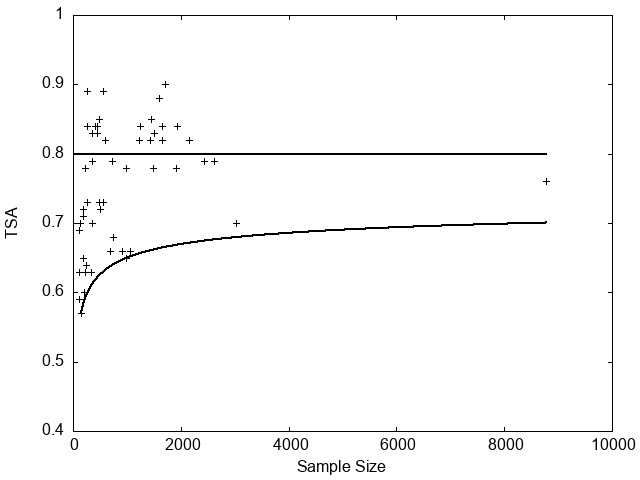

Supplement: vbag008_Supplementary_Data [file vbag008_supplementary_data.zip › acc.png]

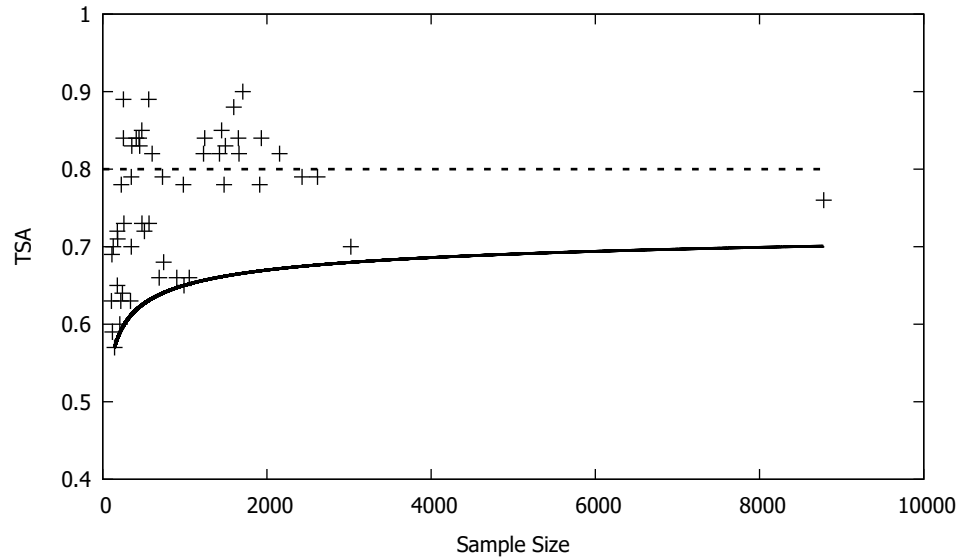

Supplement: vbag008_Supplementary_Data [file vbag008_supplementary_data.zip › acc.pdf]
